# Insight Into the Mechanism of Exercise Preconditioning in Ischemic Stroke

**DOI:** 10.3389/fphar.2022.866360

**Published:** 2022-03-08

**Authors:** Yuanhan Zhu, Yulin Sun, Jichao Hu, Zhuoer Pan

**Affiliations:** ^1^ Department of Neurosurgery, Zhejiang Rongjun Hospital, Jiaxing, China; ^2^ Department of Orthopedics, Zhejiang Rongjun Hospital, Jiaxing, China

**Keywords:** exercise preconditioning, ischemic stroke, neurprotection, apoptosis, neuroinflammation, oxidative stress

## Abstract

Exercise preconditioning has attracted extensive attention to induce endogenous neuroprotection and has become the hotspot in neurotherapy. The training exercise is given multiple times before cerebral ischemia, effectively inducing ischemic tolerance and alleviating secondary brain damage post-stroke. Compared with other preconditioning methods, the main advantages of exercise include easy clinical operation and being readily accepted by patients. However, the specific mechanism behind exercise preconditioning to ameliorate brain injury is complex. It involves multi-pathway and multi-target regulation, including regulation of inflammatory response, oxidative stress, apoptosis inhibition, and neurogenesis promotion. The current review summarizes the recent studies on the mechanism of neuroprotection induced by exercise, providing the theoretical basis of applying exercise therapy to prevent and treat ischemic stroke. In addition, we highlight the various limitations and future challenges of translational medicine from fundamental study to clinical application.

## Introduction

Stroke is primarily divided into hemorrhagic (intracranial hemorrhage and subarachnoid hemorrhage) and ischemic stroke. Ischemic stroke accounts for up to 80% of all strokes and is one of the most fatal global diseases with rapid onset, high mortality, and high disability [Amarenco et al., 2009; Hsieh et al., 2010 (accessed on 18 January 2022)]. The treatment principle behind ischemic stroke is to rapidly reconstruct blood reperfusion, restore oxygen supply to the brain, and remove harmful metabolites to reduce the cerebral infarction volume (Bhatia et al., 2010; Diprose et al., 2021). In recent years, neuroprotective agents have been studied based on anti-oxidation, anti-apoptosis, inhibition of excitatory amino acid release, anti-inflammation, vascular neuroprotection, and nanoparticles (Subedi and Gaire, 2021a; Chen et al., 2021; Zheng et al., 2021; Kaur and Sharma, 2022). However, most effective drugs in animal experiments often fail in clinical trials (Gladstone et al., 2002; Wahlgren and Ahmed, 2004). Therefore, finding other effective treatments besides drugs has been the emerging idea.

Ischemia tolerance has attracted wide attention as an effective protective strategy for cerebral ischemia. Ischemic preconditioning refers to tissue tolerance during long-term ischemic injury after one or more transient ischemia-reperfusion. It usually manifests as reduced cellular death, decreased cerebral infarct size, and improved organ dysfunction (Liu et al., 2021a; Correia et al., 2021; Ripley et al., 2021). Ischemic preconditioning is an effective neuroprotective method of endogenous cerebral ischemia, with exercise preconditioning being an essential type. Exercise preconditioning can effectively induce ischemia tolerance, exert neuroprotective effects, and alleviate brain damage post-stroke by providing training multiple times before ictus. Compared with other preconditioning methods, its advantages are easy to master, operate clinically, and easily accepted by patients (Egan et al., 2014). Moreover, clinical and animal experiments have ascertained the neuroprotective effect of exercise preconditioning Table 1. The underlying mechanism involves regulating the inflammatory response, inhibiting oxidative stress and apoptosis, promoting neural regeneration, contributing to brain structure and function remodeling, and reducing tissue injury after cerebral ischemia (Sakakima, 2019; Hafez et al., 2020; Hafez et al., 2021).

**TABLE 1:** Summary of pre-clinical studies of exercise preconditioning in ischemic stroke.

Exercise type	Exercise manner	Species and model	Outcome	Involved signal	References
treadmill exercise	10 min/day (15–25 m/min), 5 days/week for 3 weeks	male Sprague–Dawley rats, 60 min of MCAO	reduced infarct volume and ameliorated sensorimotor function	upregulate BDNF, HIF-1α, and P2X7 receptor	Otsuka et al. (2021a)
treadmill exercise or swimming	Swim or run (15 m/min) 30 min/day, 5 days/week for 3 weeks	male Wistar rats, 30 min of MCAO	Increase brain trophic support and reduce brain damage	Increase the gene expressions of TrkB, TNF-α, and MMP2	Teymuri Kheravi et al. (2021)
treadmill exercise	4 weeks, the distance of exercise per week is about 5,000 m	male Sprague-Dawley rats, 90 min of MCAO	improve neurocognitive function	Increase the basal dopamine level	Fan et al. (2021)
treadmill exercise	25 min/day for 4 days, break for 2 days, and one acute bout for 30 min	male Wistar rats, embolic stroke model	reduce the neurovascular injury and improved functional outcomes	Increase the expression of peNOS and pAMPK	Hafez et al. (2020)
treadmill exercise	30 min/day (2 m/min for the first 5 min, 3 m/min for the next 5 min, 5 m/min for the last 20 min) for 4 weeks	male Wistar rats, bilateral common carotid arteries occlusion	ameliorate shot-term memory impairment and prevent microvascular injury in the hippocampus	prevente the reduction of ZO-1 in the hippocampus and inhibite the activation of MMP-9	Lee et al. (2019)
treadmill exercise	30 min (20 m/min), 30 min (30 m/min) and 60 min (30 m/min) for 1 week each	male Sprague-Dawley rats, MCAO	attenuate neurological injury	preserve old and newly formed HSP72-containing neurons	Wang et al. (2019b)
treadmill exercise	30 min/day (25 m/min) for 3 or 5 days/week for 3 weeks	male Sprague-Dawley rats, 60 min of MCAO	reduce infarct volumes, improve neurological scores and sensorimotor function	reduce the Bax/Bcl-2 ratio and caspase-3 activation	Terashi et al. (2019)
treadmill exercise	30 min/day (25 m/min) for 5 days/week for 3 weeks	male Sprague-Dawley rats, 60 min of MCAO	reduce ischemic neuronal cell death, induce neuron- and astrocyte-mediated brain ischemic tolerance	Increase expression of HIF-1α, and inhibit 14-3-3γ/p-β-catenin Ser37 anti-apoptotic pathway	Otsuka et al. (2019)
treadmill exercise	30 min/day for 5 days/week for 8 weeks	male Wistar rats, 60 min of MCAO	improve neurological function and BBB integrity	develop higher levels of cortical VEGF-A and striatal VEGF-R2	Rezaei et al. (2018)
treadmill exercise	40 min/day (18 m/min) for 5 days/week for 4 weeks	ovariectomized mice, permanent MCAO	diminish infarct volume, and improve neurological deficits	Decrease MMP-9, and increase IL-10	Naderi et al. (2018)
treadmill exercise	5 days/week for 4 weeks, time and intensity increase progressively	male wistar rats, 60 min of MCAO	reduce brain edema and decrease the neurological movement disorders	none	Shamsaei et al. (2015)
treadmill exercise	30 min/day (15 m/min) for 3 days/week for 4.5 weeks	male C57Bl/6 mice, 13 min of global cerebral ischemia	forced treadmill exercise induce a stress response, and lead to increased neuronal damage	Increase levels of NLRP3, galectin-3, IFNγ and IL-10	Svensson et al. (2016)
treadmill exercise	30 min/day (20 m/min) for 6 days/week	male Sprague Dawley rats, 90 min of MCAO	reduce brain infarct volume and neurological deficits	Increase SOD activity and decrease the concentration of MDA	Feng et al. (2014)
treadmill exercise	30 min/day (15 m/min) for 6 days/week for 3 weeks	male Sprague Dawley rats, 120 min of MCAO	improve neurological deficits, reduce infarct volume, mitigate pathological damage in the ischemic cortex	regulation of the TLR4/NF-κB signaling pathway and the inhibition of central and peripheral inflammatory cascades	Zhu et al. (2016)
treadmill exercise	30 min/day (25 m/min) for 5 days/week for 3 weeks	male Sprague Dawley rats, 60 min of MCAO	reduce neuronal apoptosis, oxidative stress, and infract volume, ameliorate motor function, increase astrocyte proliferation and angiogenesis	enhance expression of MK and BDNF	Otsuka et al. (2016)
treadmill exercise	30 min (20 m/min), 30 min (30 m/min) and 60 min (30 m/min) for 1 week each	male Sprague Dawley rats, 90 min of MCAO	attenuate brain infarct, glial apoptosis, and neurological deficits	Increase the numbers of both the HSP20-containing neurons and the HSP20-containing glia	Lin et al. (2015)
swimming	60 min/day for 6 days/week for 4 weeks	Sprague Dawley rats, 120 min of MCAO	reduce infarct volume	upregulate the expression of HIF-1α	Wang et al. (2015)
treadmill exercise	30 min/day (20 m/min) for 6 days/week for 3 weeks	male Sprague Dawley rats, 120 min of MCAO	reduce brain infarct volume, cerebral edema and neurological deficits	regulation of PKC-α-GLT-1-Glutamate and PI3K/Akt-GLT-1-Glutamate signal pathway	Wang et al. (2014)
treadmill exercise	30 min/day (20 m/min) for 5 days/week for 2 weeks	male Sprague Dawley rats, 120 min of MCAO	improve CBF and neurologic deficits, reduce infarct volume	Decrease ET-1 expression	Zhang et al. (2013)
treadmill exercise	30 min/day (18 m/min) for 5 days/week for 3 weeks	male wistar rats, 10 min of 4-vessel occlusion model	improve behavioral functions and maintain more viable cells in the dorsal hippocampus	none	Tahamtan et al. (2013)
treadmill exercise	30 min/day (30 m/min) for 5 days/week for 3 weeks	male Sprague Dawley rats, 120 min of MCAO	reduce neurological deficit and infarct volume, increase the rates of glucose metabolism	reduce ADP/ATP ratio, increase GLUT1, GLUT3, and PFK	Dornbos et al. (2013)
treadmill exercise	30 min/day (30 m/min) for 5 days/week for 3 weeks	Sprague Dawley rats, MCAO	reduce neuronal apoptosis	inhibit the expression of MMP-9 and ERK1/2 expression	Chaudhry et al. (2010)
treadmill exercise	30 min/day (30 m/min) for 5 days/week for 3 weeks	Sprague Dawley rats, MCAO	diminish neuronal injury, reduce infarct volume	upregulate HSP-70, ERK 1/2 and Bcl-x(L), downregulate Bax and AIF	Liebelt et al. (2010)
treadmill exercise	30 min/day (30 m/min) for 5 days/week for 3 weeks	male Sprague Dawley rats, 120 min of MCAO	Decrease neurological deficits, infarct volume and leukocyte infiltration	Reduce TNF-α, ERK 1/2, MMP-9 and ICDM-1 expression	Curry et al. (2010)

BBB, blood-brain barrier; BDNF, brain-derived neurotrophic factor; CBF, cerebral blood flow; ERK1/2, extracellular signal-regulated kinase one and 2; GLT-1, glutamate transporter-1; HIF-1α, hypoxia-inducible factor-1α; HSP, heat shock protein; ICDM-1, intercellular adhesion molecule-1; MCAO, middle cerebral artery occlusion; MDA, malondialdehyde; MK, midkine; MMP, matrix metalloproteinase-9; NF-κB, nuclear transcription factor-κB; NLRP3, nucleotide-binding oligomerization domain-like receptor containing pyrin domain 3; peNOS, phosphorylated endothelial nitric oxide synthase; SOD, superoxide dismutase; TLR4, toll-like receptor-4; TNF-α, tumour necrosis factor-α; TrkB, tropomyosin receptor kinase B; VEGF-A, vascular endothelial g PKC-α, protein kinase C-α; rowth factor A; VEGF-R2, vascular endothelial growth factor receptor 2; ZO-1, zonula occludens-1.

## Mechanism of Exercise Preconditioning Induced Cerebral Ischemia Tolerance

### Attenuation of Neuronal Apoptosis

Apoptosis is programmed cell death, having the characteristics of selectivity, initiative, and reversibility. Cellular necrosis is characterized by cell swelling, membrane rupture, and random degradation of DNA. In contrast, cellular apoptosis involves dense chromatin, formation of DNA fragments, cytoplasmic foam, and apoptotic bodies (Park et al., 2021a; Moujalled et al., 2021; Saleem, 2021). Apoptosis is crucial in ischemic injury and is the primary form of delayed neuronal death after cerebral ischemia (Mitsios et al., 2007; Radak et al., 2017; Uzdensky, 2019). Therefore, brain damage will be alleviated if the occurrence and development of neuronal apoptosis are effectively prevented. Primarily, there are three apoptotic pathways: endoplasmic reticulum stress pathway, death receptor pathway, and mitochondrial pathway (Prentice et al., 2015; Redza-Dutordoir and Averill-Bates, 2016; Wei et al., 2018). In addition, many apoptosis-related genes and proteins are regulated and involved in apoptosis after cerebral ischemia (Ferrer et al., 2003; Uzdensky, 2019).

Previous studies have observed that exercise preconditioning can effectively alleviate cerebral ischemia associated tissue damage caused. One study revealed that preconditioned exercise retained more surviving neurons within the hippocampus of the ischemic brain tissue, effectively reducing neuronal death (Tahamtan et al., 2013). Another report depicted that exercise training could effectively induce autophagy and reduce neuronal apoptosis after stroke (Zhang et al., 2013). Exercise can induce the expression of the heat shock protein (HSP)-70, which attenuates apoptosis by inhibiting apoptosis-inducing factors and elevating anti-apoptotic proteins expression, such as Bcl-2, leading to the alleviation of cerebral ischemic injury (Zhang et al., 2011). Wang et al. (2019b) observed that preischemic treadmill exercise improves post ischemic brain injury outcomes by preserving both the old and newly formed HSP-72-containing neurons within rats. Similarly, Lin et al. (2015) proposed that preischemic treadmill exercise improves the outcome of ischemic stroke by elevating the numbers of neurons and glial cells containing HSP-20. In addition, several studies explored the potential mechanism underlying exercise-induced neuroprotection after ischemic stroke. Liebelt et al. (2010) suggested that exercise preconditioning can reduce neuronal apoptosis and cerebral infarction volume through upregulation of HSP-70 and ERK ½. Additionally, ERK and HSP-70 inhibitors could simultaneously eliminate the protective effects of exercise preconditioning on the brain. Other studies found that preischemic treadmill exercise reduced hippocampal microvascular injury after stroke, prevented zonula occludens-1 reduction in the hippocampus, and inhibited matrix metalloproteinase-9 (MMP-9) activation after stroke (Lee et al., 2019). Another team also revealed the changes of MMP-9 in stroke mice, and they observed that exercise preconditioning induced a better outcome than the control ischemic mice, manifested by reduced MMP-9, diminished infarct volume, and significantly improved neurological deficits (Naderi et al., 2018). Exercise preconditioning may inhibit MMP-9 activity by upregulating ERK1/2 expression and reducing neuronal apoptosis level after cerebral ischemia (Chaudhry et al., 2010). ERK-mediated signaling pathways are involved in ischemia-induced apoptosis and regulate Bax and Bcl-2 protein expression after stroke (Li et al., 2021b). The mechanism of exercise preconditioning affecting Bcl-2 and Bax proteins expression is similar to hypoxia preconditioning, among which caspase 3, Bcl-2, and Bax are the core members regulating neuronal apoptosis (Liu et al., 2021d). Choi et al. (2013) observed that short-term running exercises inhibited the division of DNA induced by hypoxic-ischemic injury. Thus, it effectively reduced the expression of caspase-3 and inhibited neuronal apoptosis (Choi et al., 2013). Zhang et al. (2019). showed that voluntary wheel running inhibits cellular apoptosis by downregulating the Bax/Bcl-2 ratio and caspase-3 protein expression. On further analysis, both mild exercise postconditioning and intense exercise postconditioning significantly decreased brain infarct volumes and apoptosis compared to the resting rats. Moreover, mild exercise postconditioning enhanced Bcl-2 expression and the Bcl-2/Bax ratio (Li et al., 2021a). Controversially, Li et al. (2017b) found that Bcl-2 expression was not affected by exercise after stroke, indicating the importance of the exercise time point. Terashi et al. (2019) investigated the neuroprotective effect of various frequency preconditioning exercises on neuronal apoptosis post cerebral ischemia in rats. They observed that high-intensity preconditioning exercise for three or more times per week exert neuroprotective effects by downregulating the Bax/Bcl-2 ratio and caspase-3 activation after stroke (Terashi et al., 2019). The above mentioned results indicate that both pre- or postconditioning exercise can potentially induce ischemic tolerance by regulating apoptosis and anti-apoptosis-related proteins. Therefore, exploring the most suitable time points, intensity and frequency of exercise should be incorporated in future studies.

### Inhibition of Oxidative Stress

When the body is subjected to harmful stimulation, the oxidation-antioxidation balance system is broken, leading to oxidative tissue damage through the accumulation of reactive oxygen species (ROS) in cells (Lushchak et al., 2021). ROS mainly includes singlet oxygen, ozone, hydrogen peroxide, and oxygen-free radicals. ROS can be produced through aerobic metabolism during normal physiological conditions, and the production and elimination of ROS maintain a dynamic balance in the body. Nitricoxidesynthas, cyclooxygenase, xanthine dehydrogenase/xanthine oxidase, reduced-type coenzyme II oxygenase, myeloperoxidase, and other enzymes promote ROS production. In contrast, superoxide dismutase, catalase, peroxidase, glutathione peroxidase, and other enzymes inhibit ROS production (Kalyanaraman, 2013; Griffiths et al., 2014; Moldogazieva et al., 2018). Increased oxygen free radical generation and/or decreased scavenging capacity of the anti-oxidation system in the injured area after cerebral ischemia contributes to ROS (Shao et al., 2020; Duan et al., 2021; Jelinek et al., 2021), leading to neuronal death (Li et al., 2018). Brain tissue is rich in lipids and is highly sensitive to oxidative damage caused by ROS, characterizing oxidative stress as an essential target in treating ischemic stroke (Liu et al., 2002; Liu, 2003; Schönfeld and Reiser, 2017).

Ostuka et al. (2021b) conducted an animal study investigating the role of exercise preconditioning in subarachnoid hemorrhage (SAH). It was found that preconditioning ameliorates early brain injury post SAH. Moreover, the expression of 4-hydroxynonenal and nitrotyrosine was reduced by Nrf2/HO-1 pathway activation, improving the oxidative stress indicators (Otsuka et al., 2021b). Another study from the same team revealed that exercise preconditioning could decrease ROS in focal brain ischemia (Otsuka et al., 2016). Leite et al. (2012) found that swim training could relieve oxidative damage under metabolic stress by inhibiting glutamic acid and promoting the release of nitric oxide. In addition, several animal studies have also established that exercise preconditioning can effectively reduce oxidative damage of brain tissue during cerebral ischemia-reperfusion. Long-term and short-term exercise preconditioning can elevate antioxidant enzyme levels in the hippocampus and cortex, reduce the malondialdehyde content, inhibit oxidative stress, thereby alleviating oxidative damage post cerebral ischemia-reperfusion. This effect was coupled with improved sensory-motor function and memory. Therefore, it suggests that reducing oxidative stress could be an essential mechanism of exercise preconditioning-induced cerebral ischemia tolerance (Radak et al., 2007; Schimidt et al., 2014; Sosa et al., 2015; Chrishtop et al., 2020). The combination therapy of exercise and scalp acupuncture counteracts ischemic brain injury through ROS downregulation, suggesting a potential therapeutic approach in stroke patients (Li et al., 2020b).

Hypoxia inducible factor-1α (HIF-1α) is a sensitive oxygen homeostasis regulator and can be rapidly induced by hypoxia/ischemia. It plays a vital role in ischemic stroke through various mechanisms, including oxidative stress regulation, apoptosis, inflammation, and angiogenesis (Guglielmotto et al., 2009; Miyata et al., 2011; Cheng et al., 2014; Jiang et al., 2020; Peng et al., 2020; Zhang et al., 2021a; He et al., 2021). Previous studies have also determined that HIF-1α is crucial in ischemic preconditioning, which reduces brain damage post cerebral ischemia (Liu et al., 2005). HIF-1α exhibits beneficial effects mediated by the Akt signaling pathway and neuroinflammatory response multi-modulation in remote ischemic preconditioning (Yang et al., 2018; Du et al., 2020). In addition, upregulation of HIF-1α expression by hypoxic preconditioning promotes angiogenesis and neurogenesis. It reduces neuronal death and improves neurological function post ischemic stroke (Chen et al., 2017). Moreover, HIF-1α is involved in attenuating hyperglycemia-enhanced hemorrhagic transformation through MMP-2 and MMP-9 inhibition post-stroke (Soejima et al., 2013). As one of the crucial ways of ischemic preconditioning, exercise-induced neuroprotection is significantly associated with HIF-1α. Exercise preconditioning enhanced HIF-1α expression, contributing to elevated glucose metabolism and ATP production rates after ischemic stroke (Dornbos et al., 2013). Furthermore, exercise preconditioning stimulates the release of HIF-1α. It enhances neurogenesis and angiogenesis (Li et al., 2017a), promoting synaptic plasticity (Li et al., 2020a), and reducing neuronal apoptosis (Otsuka et al., 2019). However, exercise preconditioning-induced neuroprotective effect could be quickly lost after exercise cessation. This outcome is a reminder that regulating HIF-1α expression in a time-dependent manner may potentially focus on the further treatment of ischemic stroke (Otsuka et al., 2021a).

### Suppression of Inflammation

An inflammatory response is a pivotal part of the pathological process of ischemic brain injury. The inflammatory response involves a series of inflammatory cells and mediators, which have a dual effect of damage and repair in the occurrence and development of cerebral ischemia. Its effect is correlated with time, scope, and the severity of inflammation (Ceulemans et al., 2010; Wang et al., 2019a; Pluta et al., 2021). Studies have shown that inflammation factor expression in the ischemic region increased significantly within a few hours after cerebral ischemia, with tissue damage caused by various mechanisms, including microvascular occlusion, oxygen free radical generation cytotoxicity enzyme, and chemokine release (Zhang et al., 2021b; Ma et al., 2021).

Glial cells are a significant group of cells in the brain. The number of glial cells is 10–50 times that of neurons and has almost the same total volume as that of neurons. They are mainly categorized into astrocytes, oligodendrocytes, and microglias (Xu et al., 2020; Sancho et al., 2021). Microglia secretes inflammatory molecules at the injury site to protect healthy neurons and remove the dead ones. During cerebral ischemia, microglia are rapidly activated, presenting antigens, and releasing inflammatory factors like IL-1β, IL-6, and TNF-α. In contrast, during the recovery stage of the brain, microglia exhibits an anti-inflammatory role (Zhang, 2019; Berchtold et al., 2020; Kang et al., 2020; Subedi and Gaire, 2021b; Hou et al., 2021). Many scholars have explored the impact of microglia during exercise. High-intensity interval training elicited better responses at functional and cardiovascular levels than moderate-intensity continuous training after ischemic stroke. Thus, inflammasome-mediated pyroptosis could be suppressed by the anti-inflammatory effect of exercise due to the shifting of microglial polarization towards the neuroprotective M2 phenotype (Liu et al., 2021b). Moreover, treadmill exercises improved short-term memory, inhibited reactive astrogliosis and microglial activation, and suppressed the expression of adhesion molecules and pro-inflammatory cytokines in hyperlipidemic rats (Park et al., 2021b). Casaletto et al. (2022) supported the conclusion that physical activity could be leveraged to reduce pro-inflammatory microglial states in humans through modifiable behavior. They monitored physical activities and cognitive performances in life and quantified the microglial activation and synaptic markers inside brain tissue at death (Casaletto et al., 2022). Treadmill exercise can significantly ameliorate cerebral ischemia-reperfusion injury through IL-4 expression elevation to promote M2 microglia polarization through the JAK1-STAT6 pathway (Lu et al., 2021).

Astrocytes are the most abundant cell type in the central nervous system responding to various disease states. They assist in clearing excessive potassium ions around neurons by regulating the osmotic balance of ions and water and maintaining the relative stability of the neuronal external environment (Jensen et al., 2013; Dinuzzo et al., 2017; Yang et al., 2021b). Astrocytes are also involved in the inflammatory response post cerebral ischemia (Gao et al., 2021; Mi et al., 2021; Kieran et al., 2022), although their roles are different in different stages of inflammation. In the initial phase of inflammation, astrocytes behave as antigen-presenting cells and secrete pro-inflammatory antigen-presenting cytokines to protect tissues from damage. During the inflammatory response and repair phase peak, astrocytes act as inflammatory regulatory cells, secreting anti-inflammatory cytokines and promoting tissue repair (Regunathan and Piletz, 2003). Jiang et al. (2021) investigated the physical exercise influence on activated astrocytes polarization. They observed that the impact of physical exercise on white matter repair and cognition improvement could be related to astrocytes polarization regulation, inducing myelin debris clearance and efficient remyelination (Jiang et al., 2021). He et al. (2017) revealed that voluntary wheel running accelerated glymphatic clearance, improved the expression and polarization of astrocytic aquaporin 4, attenuated neuroinflammation, and protected mice against synaptic dysfunction and decline in spatial cognition. In addition, Sun et al. (2018) observed that physical exercise released the immune response by decreasing cytokine levels and astrocytes population. Voluntary physical training could modulate the reactive astrocyte state, linked through astrocytic brain-derived neurotrophic factor (BDNF) to improve hippocampal cognition (Belaya et al., 2020).

### Promotion of Neurogenesis

Traditionally, the non-regeneration of neurons is the main reason for the difficulty in neurological functional recovery (Caleo, 2015; Jones, 2017). Recently, researchers have identified that neurons have plasticity and the ability to repair post-injury, which can reshape nerve functions after ischemic stroke. Studies have found that ischemia-induced brain injury can be attenuated by regenerating neurons, synapses, and vessels, improving the defense capability of brain tissue. Moreover, the blood supply to the ischemic area can be restored, thereby promoting remodeling of neural function after ischemic injury (Yang et al., 2021a; Liu et al., 2021c; Zong et al., 2021; Puderbaugh and Emmady, 2022). The improved outcomes indicate that neural regeneration is an essential mechanism behind exercise preconditioning inducing ischemia tolerance (Shamsaei et al., 2015). Praag et al. observed that voluntary exercise ameliorates certain deleterious morphological and behavioral consequences of aging connected with neurogenesis regulation (van Praag et al., 2005). Another study found that treadmill exercise improved short-term and spatial memories by elevating neurogenesis and suppressing apoptosis within the hippocampal dentate gyrus of old-aged rats (Kim et al., 2010). Codd et al. revealed that elevated neurogenesis is sufficient to reverse hippocampal injury-induced deficits in either the damaged or intact hippocampus (Codd et al., 2020). Moreover, the improvement in hippocampal-based learning in aged mice after physical exercise is dependent on neurogenesis in the dentate gyrus and is regulated by growth hormone level changes. Specific changes in hippocampal circuitry underlying the cognitive improvements resulting from physical activity were also identified, suggesting dependency on neurogenesis activation in aged animals (Blackmore et al., 2021; Zhou et al., 2021). Cheng et al. (2020) observed that treadmill exercise promotes neurogenesis and myelin repair by upregulating the Wnt/β-catenin signaling pathway and improves the neurological deficit caused by focal cerebral ischemia/reperfusion. Similarly, Hong et al. (2020) showed that treadmill exercise enhanced motor function and short-term memory by elevating synaptic plasticity and neurogenesis in thrombotic stroke mice. Zhang et al. (2020) indicated that post-stroke exercise improved behavioral function recovery, where synaptogenesis was a beneficial factor.

BDNF plays a vital role in increasing synaptic plasticity and promoting neural regeneration. Xu et al. (2021) found an upregulation of BDNF and TrkB in the treadmill exercise group in rats. BDNF/TrkB signaling pathway could modulate the impact of exercise and the enriched environment by improving learning and memory in rats. BDNF expression levels in the ischemic brain were significantly upregulated post exercise cessation in an animal study (Wang et al., 2020), consistent with another study (Xu et al., 2021). Interestingly, a meta-analysis summarized the effects of physical exercise with different intensities, duration, and frequency on peripheral BDNF levels among the sedentary elderly without any cognitive impairment. The results showed that physical exercise did not cause any significant difference in peripheral BDNF concentration (Fleitas et al., 2022), which indicates that BDNF expression in the brain and peripheral plasma are influenced differentially by exercises.

## Prospects

**FIGURE 1:**
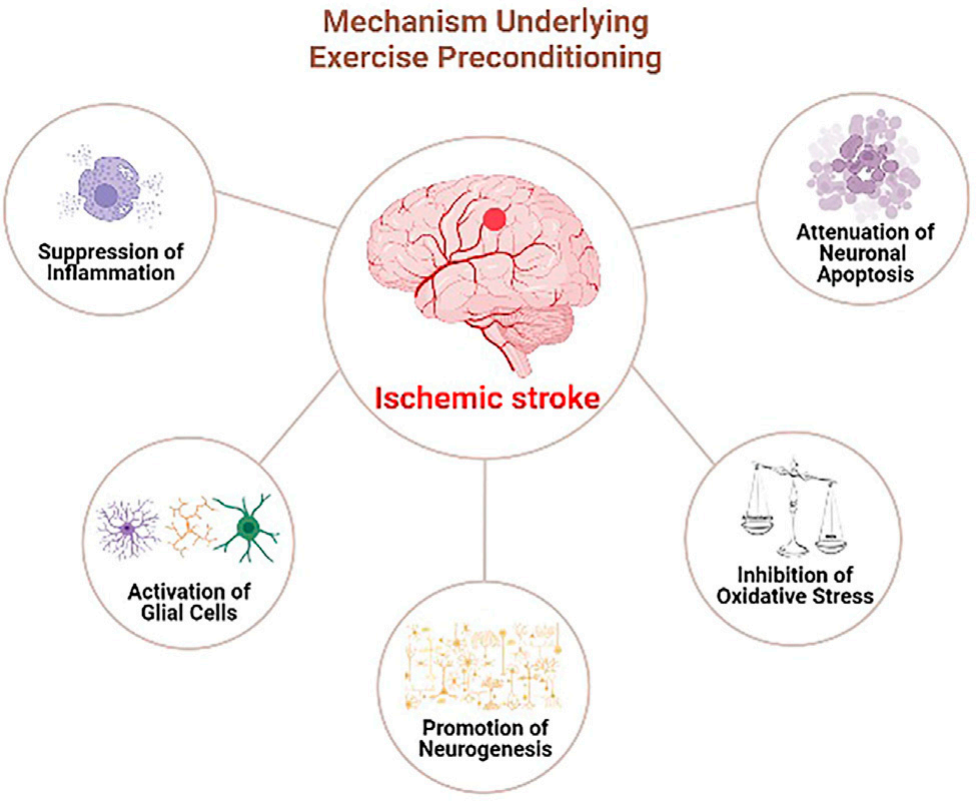
The involved mechanisms underlying preconditioning exercise-induced neuroprotection in ischmeic stroke.

Therefore, exercise preconditioning could induce ischemia tolerance by inhibiting neural apoptosis and oxidative stress, regulating the inflammatory response, promoting neural regeneration, and exerting preventive and protective effects on the ischemic brain injury (Figure 1). Exercise preconditioning depicts a significant application prospect being a safe and slight side-effect strategy to prevent cerebral ischemia. Further studies on the neuroprotective mechanism of exercise preconditioning will identify new therapeutic targets for ischemic stroke. Moreover, supporting exercise training could provide a solid theoretical foundation as effective prevention and control measures of ischemic stroke patients.

However, many problems regarding exercise preconditioning require attention. First, the heterogeneity of population subgroups, including age, gender, dietary habits, etc., should be considered. Different hypoxic degrees, duration, and intensity will induce different effects. For example, how does exercise play a neuroprotective role in inducing cerebral ischemia tolerance among the elderly population with the most incidence of ischemic stroke? What type of exercise, frequency, intensity, and duration could harness the best results? Second, there is a lack of specific indicators to analyze the effect of exercise preconditioning. Applying mild stress may exacerbate the disease state rather than provide a cure in some disease cases. This outcome necessitates understanding the preconditioning and ischemic stroke mechanisms and the stress response of cells/tissues/organs at different stages of ischemic stroke. Moreover, it also requires searching for specific physiological biomarkers to improve the monitoring of disease progression or treatment effectiveness. In addition, the exercise preconditioning mechanism needs to be further explored. Does exercise directly affect the brain or protect brain function through peripheral effect? Which group of brain cells is more sensitive to exercise stimulation? Finally, combining exercise preconditioning with traditional medicine, nanomedicine, or other preconditioning methods needs to be studied, which could be a potential therapeutic approach for ischemic stroke.
